# Development and validation of a selenium metabolism regulators associated prognostic model for hepatocellular carcinoma

**DOI:** 10.1186/s12885-023-10944-w

**Published:** 2023-05-18

**Authors:** Huishan Sun, Junyu Long, Bangyou Zuo, Yiran Li, Yu Song, Minghang Yu, Ziyu Xun, Yanyu Wang, Xi Wang, Xinting Sang, Haitao Zhao

**Affiliations:** 1 Department of Liver Surgery, State Key Laboratory of Complex Severe and Rare Diseases, Peking Union Medical College Hospital, Chinese Academy of Medical Sciences & Peking Union Medical College, Beijing, China; 2 Medical Research Center, State Key Laboratory of Complex Severe and Rare Diseases, Peking Union Medical College Hospital, Chinese Academy of Medical Science and Peking Union Medical College, Beijing, China; 3 Department of Hepatobiliary Surgery, Sichuan Academy of Medical Sciences & Sichuan Provincial People’s Hospital, School of Medicine, University of Electronic Science and Technology of China Chengdu, Sichuan, China; 4 Department of Allergy, Beijing Tongren Hospital, Capital Medical University, Beijing, China; 5 Beijing Institute of Infectious Diseases, Beijing, China; 6 Institute of Infectious Diseases, Beijing Key Laboratory of Emerging Infectious Diseases, Beijing Ditan Hospital, Capital Medical University, Beijing, China

**Keywords:** Hepatocellular carcinoma, Selenium, Metabolism, Prognosis, INMT

## Abstract

**Background:**

Selenium metabolism has been implicated in human health. This study aimed to identify a selenium metabolism regulator-based prognostic signature for hepatocellular carcinoma (HCC) and validate the role of INMT in HCC.

**Methods:**

Transcriptome sequencing data and clinical information related to selenium metabolism regulators in TCGA liver cancer dataset were analysed. Next, a selenium metabolism model was constructed by multiple machine learning algorithms, including univariate, least absolute shrinkage and selection operator, and multivariate Cox regression analyses. Then, the potential of this model for predicting the immune landscape of different risk groups was evaluated. Finally, INMT expression was examined in different datasets. After knockdown of INMT, cell proliferation and colony formation assays were conducted.

**Results:**

A selenium metabolism model containing INMT and SEPSECS was established and shown to be an independent predictor of prognosis. The survival time of low-risk patients was significantly longer than that of high-risk patients. These two groups had different immune environments. In different datasets, including TCGA, GEO, and our PUMCH dataset, INMT was significantly downregulated in HCC tissues. Moreover, knockdown of INMT significantly promoted HCC cell proliferation.

**Conclusions:**

The current study established a risk signature of selenium metabolism regulators for predicting the prognosis of HCC patients. INMT was identified as a biomarker for poor prognosis of HCC.

**Supplementary Information:**

The online version contains supplementary material available at 10.1186/s12885-023-10944-w.

## Background

Hepatocellular carcinoma (HCC) is a highly malignant cancer and was the third leading cause of death worldwide in 2020, with approximately 906,000 new cases and 830,000 deaths [1]. The incidence rate of HCC is extremely high in China, with approximately 370,000 new cases in China in 2015 [2]. Many risk factors, including hepatitis B virus (HBV) and hepatitis C virus (HCV), can influence the tumorigenesis of HCC [3]. Although current therapeutic options include surgical resection and systemic therapy, the overall survival (OS) of patients remains unsatisfactory [4].

Recently, cancer cell metabolism has been shown to play a pivotal role in tumour progression [5]. One important feature of cancer cell metabolism is the acquisition of abnormal amounts of nutrients to maintain cell viability and build new biomass [6]. In addition to the principal nutrients glucose and glutamine, various micronutrients, including trace metals and trace minerals such as selenium, contribute to tumorigenesis. Selenium is an essential trace mineral that is fundamental to human health [7]. Selenium can be not only incorporated into selenoproteins to form the rare amino acid selenocysteine [8], which is essential for the survival of cancer [9], but also methylated for the detoxification of excess selenium [10]. Thus, selenium metabolism seems to play an important role in cancer but further investigation, especially related to its role in liver cancer, is needed.

Oncogene-driven metabolic reprogramming allows cancer cells to maintain deregulated proliferation [11]. Thus, genes involved in selenium metabolism regulation also play important roles in cancer cells. Some studies have examined the expression of selenoprotein genes in colorectal adenoma, colorectal cancer [12] and breast cancer [13]. However, an integrated understanding of selenium metabolism genes in HCC, including the interactions between selenium metabolism regulators and the tumour environment, is lacking.

Our study aimed to systematically assess the correlations of selenium metabolism regulators with prognosis, immune check-point and the tumour immune microenvironment. We ultimately elucidated the role of indolethylamine n-methyltransferase (INMT) in HCC progression.

## Methods

### Data acquisition of TCGA liver cancer samples

The gene expression RNA sequencing (RNA-Seq) data of a liver hepatocellular carcinoma (LIHC) cohort, including primary tumour (cancer) and solid tissue normal (normal) samples and their corresponding clinical survival information, were downloaded from The Cancer Genome Atlas (TCGA) data portal (https://xenabrowser.net/hub/). The Liver Cancer-RIKEN-Japan (LIRI-JP) cohort data were downloaded from the International Cancer Genome Consortium (ICGC) database. Gene expression data and clinical information for the training and validation cohorts were obtained from the TCGA and LIRI-JP databases. Six HCC datasets, GSE22058, GSE25097, GSE36376, GSE46444, GSE54236 and GSE64041, were obtained from the Gene Expression Omnibus (GEO) database (https://www.ncbi.nlm.nih.gov/geo/). Gene expression profiling interactive analysis (GEPIA) according to the GEPIA2 website (http://gepia2.cancer-pku.cn/) [14] was used to compare the differential expression of selenium metabolism genes between liver tumour tissue and normal tissues.

### Selection of selenium metabolism regulators

**Fig. 1:**
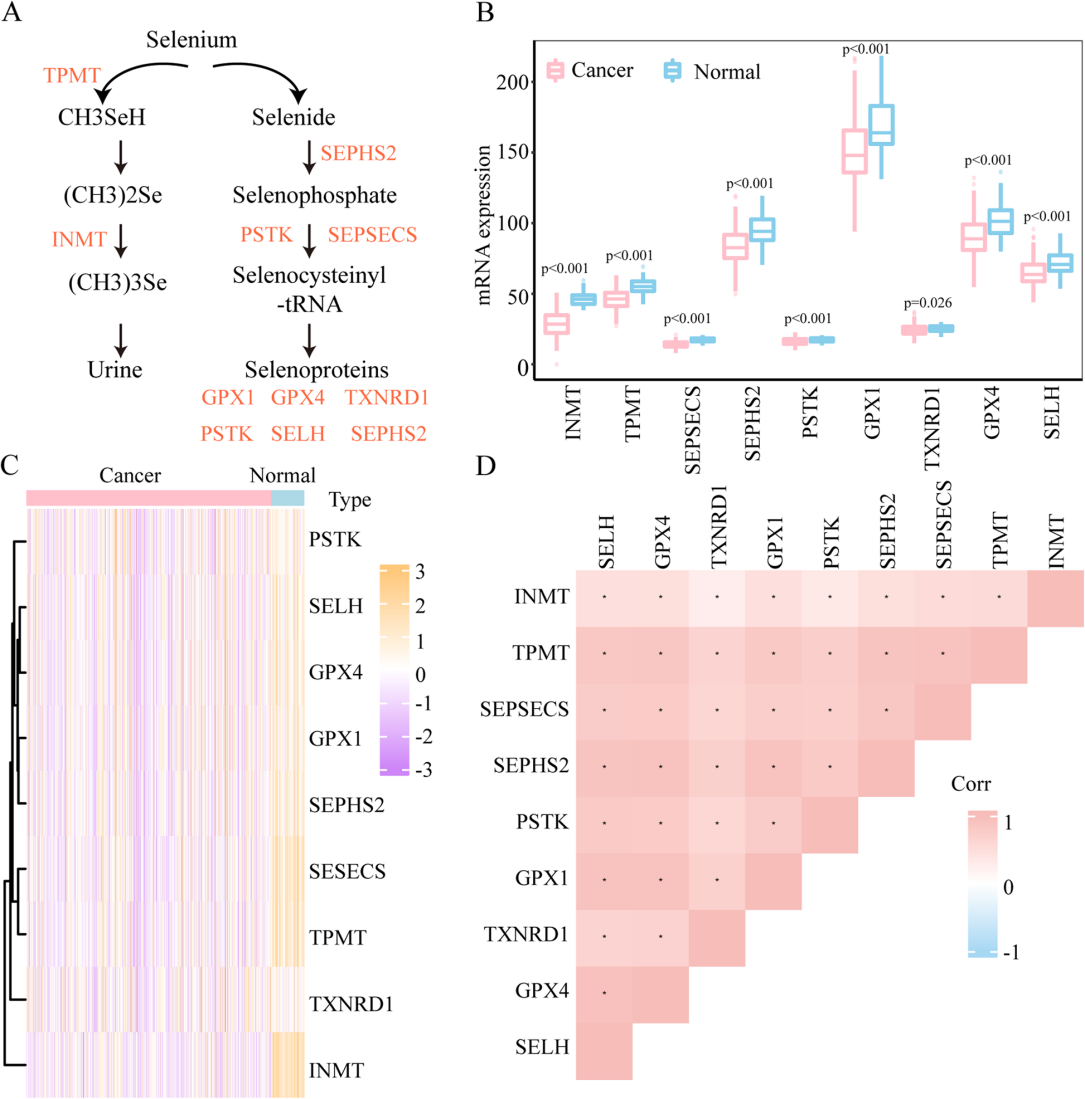
Expression characteristics and correlations of selenium metabolism regulators in hepatocellular carcinoma (HCC). **A** Diagram of the selenium metabolism pathway. **B** and **C**. The differential expression levels (**B**) and heatmap (**C**) of selenium metabolism genes in TCGA liver cancer samples (normal [n = 50] and tumour [n = 371]). (**D**) The correlations among 9 selenium metabolism regulators were analysed by Pearson correlation

Nine genes, namely, selenide water dikinase 2 (SEPHS2), l-seryl-trna(sec) kinase (PSTK), o-phosphosery-trna (sec) selenium transferase (SEPSECS), glutathione peroxidase 1 (GPX1), thioredoxin reductase 1 (TXNRD1), glutathione peroxidase 4 (GPX4), selenoprotein h (SELH) (also named C11orf31), INMT, and thiopurine s-methyltransferase (TPMT) (Fig. 1A), have been reported to regulate selenium methylation and the survival of cancer cells [9]. To completely understand the expression profiles of these nine genes in HCC, heatmap and overall expression analyses were conducted.

### Selenium metabolism model construction and validation

First, univariate Cox regression analysis was performed to determine the selenium metabolism-related genes that have an impact on prognosis (*P* < 0.01) in the training cohort. Second, the selected selenium metabolism-related genes were subjected to LASSO Cox regression analysis [15]. The coefficients of each gene from LASSO regression analysis were used to yield the following risk score equation: risk score = sum of coefficients x selenium metabolism gene expression level. Third, we used multivariate Cox regression analysis to establish a formula to calculate the risk score for each patient. Based on the highest χ2 value defined in the Mantel–Cox test, the optimum cut-off value for classifying all patients into low-risk and high-risk groups was determined by X-tile software. Finally, a validation cohort (LIRI-JP) consisting of 260 virus-associated HCC donors was used to validate the same formula and the same cut-off value as those in the training cohort.

### Independent prognostic performance of the selenium metabolism model

We performed univariate and multivariate Cox analyses to investigate whether the selenium metabolism gene model was independent of other clinicopathological factors (sex, age, and pathological stage). To allow clinicians to quantitatively predict patients’ 1-year, 3-year, and 5-year survival probabilities, a nomogram integrating the selenium metabolism gene model and numerous clinicopathological characteristics was constructed. We selected clinicopathological characteristics that were obviously associated with survival time in the univariate and multivariate analyses (*P* < 0.05) to establish the nomogram. Calibration curves were drawn to assess the degree of fit between the actual and nomogram-estimated 1-year, 3-year, and 5-year survival probabilities.

### Evaluation of immune infiltration and immune function

The gene sets of immune cells and immune functions were obtained from the study of He et al. [16]. Subsequently, the enrichment scores of each immune cell and immune function in each sample were calculated according to the single-sample gene set enrichment analysis (ssGSEA) method. Additionally, the Microenvironment Cell Populations-counter (MCP-counter) algorithm was employed to estimate the abundances of immune cells [17]. The cytolytic activity (CYT) score was determined by the geometric mean expression of perforin 1 (PRF1) and granzyme A (GZMA) [18]. The “pRRophetic” R package was used to assess drug sensitivity.

### Clinical human specimens and experimental validation

Liver cancer tissues and adjacent normal samples were collected from patients who underwent surgery and were pathologically diagnosed with HCC at Peking Union Medical College Hospital (PUMCH). All patients provided written informed consent. All procedures were performed with the permission of the Peking Union Medical College Hospital Ethics Board. RNA was extracted from fourteen paired tumour samples and adjacent normal samples to quantify INMT mRNA expression. The primer sequences were as follows: INMT forwards primer: 5’-ATCACTCTCTCCGACTTTACCG-3’, INMT reverse primer: 5’-GCTGGGGTCCAGTCATAGG-3’, actin forwards primer: 5’-GACCTGACTGACTACCTCATGAAGAT-3’, and actin reverse primer: 5’-GTCACACTTCATGATGGAGTTGAAGG-3’. Moreover, INMT protein expression was determined using six paired HCC samples. Antibodies against the following proteins were used in this study with the indicated sources, catalogue numbers, and dilutions: GAPDH (ProteinTech, Chicago, USA; 60,004–1-Ig, 1:3000) and INMT (ProteinTech, Chicago, USA; 21,578–1-AP, 1:500).

### Cell culture and transfection

LO2, Huh-7, SK-HEP-1, and HepG2 cells were cultured in Dulbecco’s modified Eagle’s medium (DMEM, Gibco). SMMC-7721 cells were cultured in standard RPMI 1640 medium (Gibco). All media were supplemented with 10% foetal bovine serum (Gibco), 100 μg/mL penicillin, and 100 U/mL streptomycin (Invitrogen). All cells were cultured in a 5% CO2 humidified incubator (Thermo Fisher Scientific, USA) at 37 °C. All the cells were routinely examined for mycoplasma contamination.siRNAs were transfected into cells using Lipofectamine RNAiMAX transfection agent (Invitrogen, Carlsbad, CA) following the manufacturer’s instructions. The following siRNAs were ordered from GenePharma Company (Suzhou, China): INMT #1, 5’-GCUUCAUUGUGGCUCGCAATT-3', and INMT #2, 5’-CAGCUAUCUUAGAUGCGAUTT-3'.

### Cell proliferation assay

Cell proliferation assays were performed as described previously [19]. In brief, cells were transfected with siRNAs for 48 h and seeded in 6-well cell culture dishes in triplicate at a density of 60,000 cells. The medium was changed every two days. The cell number at the indicated time points was calculated by counting. Cell Counting Kit-8 (CCK-8) assay was used to analyse cell viability. After INMT knockdown, almost 5000 cells were seeded into 96-well plates and the medium was changed every two days. The cells were treated with CCK-8 buffer and cell absorbance at 450 nm was measured.

### Colony formation assay

For crystal violet staining, cells were seeded in a 6-well plate for almost two weeks. Then, the cells were fixed with methanol for 30 min and stained with 0.05% crystal violet for another 30 min. After washing with PBS, the cells were photographed and counted.

### Statistical analysis

Statistical analyses were performed using R software v.4.0.3. and GraphPad Prism 8.0 software. The results are shown as the means ± standard deviations (SDs). Student’s t test and the chi-square (χ2) test were performed to evaluate differences between the two groups. Kaplan–Meier analysis was used to analyse the survival difference between the high-risk and low-risk groups. Pearson correlation analyses were performed to identify the association among the selenium metabolism genes. When the P value was < 0.05, differences were considered significant.

## Results

### The expression profile of selenium metabolism regulators in LIHC patients

Nine selenium metabolism genes, including 7 genes regulating selenium detoxification (SEPHS2, PSTK, SEPSECS, GPX1, GPX4, TXNRD1, and SELH) and two genes regulating selenium methylation (INMT and TPMT), were analysed (Fig. 1A). Compared with normal tissues, all the genes were differentially expressed in cancer tissues (Fig. 1 B and C and Supplementary Fig. 1). In the paired tumour sample analysis, the expression level of TXNRD1 in the tumour sample was the same as that in the normal sample (Supplementary Fig. 1). In addition, Pearson correlation analysis revealed that selenium metabolism genes were significantly connected with each other (Fig. 1D). Taken together, these results depict the expression profiles and correlations of selenium metabolism regulators in HCC.

### Construction and assessment of the selenium metabolism gene model in the training cohort

**Fig. 2:**
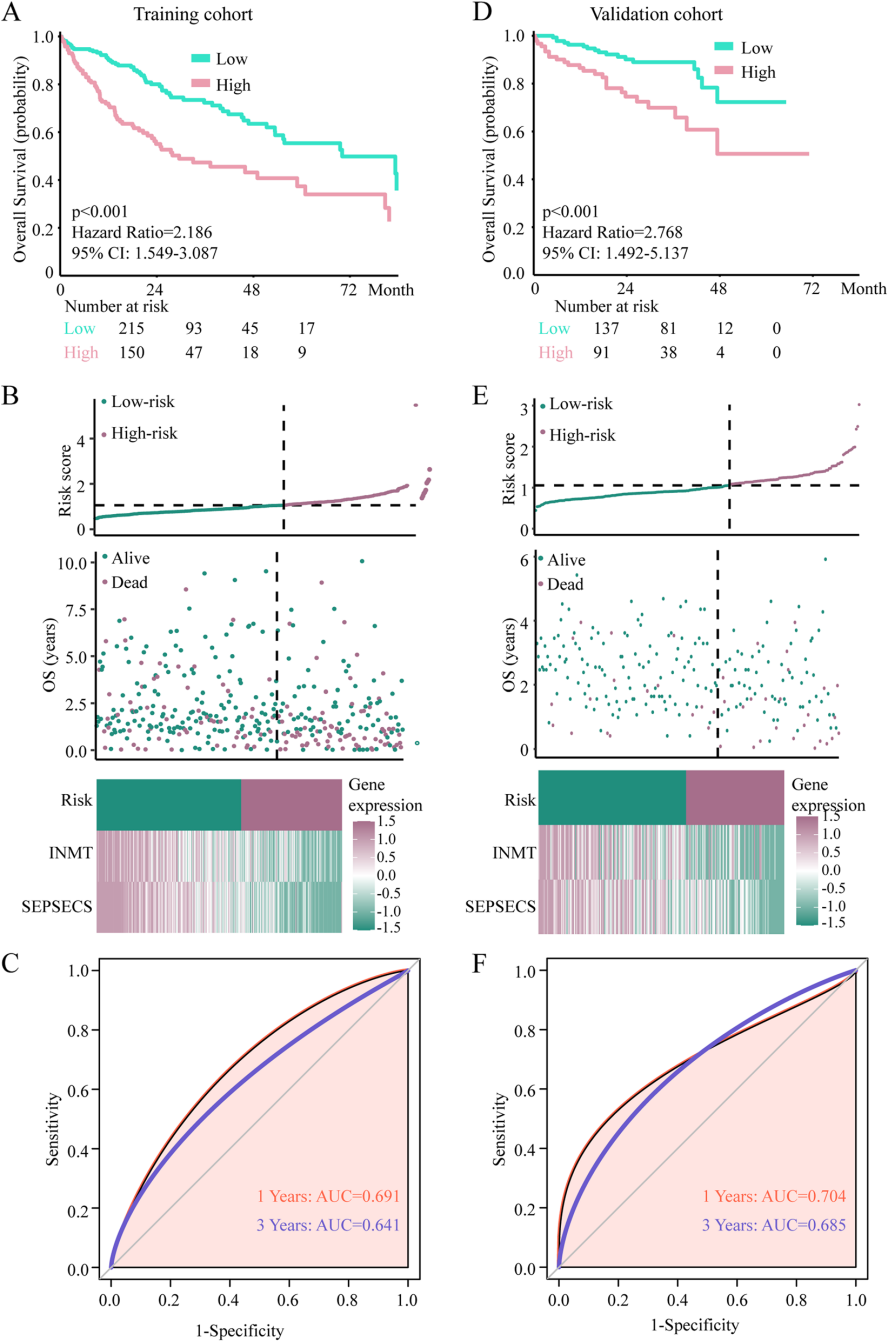
Construction and validation of the selenium metabolism model. **A** and **D** Survival analysis of the selenium metabolism model in the training cohort (**A**) and the validation cohort (**D**). **B** and **E** Distribution of the expression of selenium metabolism genes, patient survival status, and the risk score between the high-risk and low-risk groups in the training cohort (**B**) and the validation cohort (**E**). **C** and **F** ROC curves of the selenium metabolism gene-mediated model for predicting 1-year and 3-year overall survival in the training cohort (**C**) and the validation cohort (**F**)

To better understand the prognostic significance of selenium metabolism genes in LIHC patients, the nine genes were subjected to 1000 repeats of LASSO Cox regression analysis to further identify robust selenium metabolism signatures. Four genes were identified, of which only INMT and SEPSECS appeared more than 900 times of 1000 total repeats of LASSO Cox regression analysis. Finally, we used multivariate Cox regression analysis to develop a selenium metabolism model. The formula was as follows: risk score = (-0.23 × SEPSECS expression level) + (-0.19 × INMT expression level). This result indicated that higher expression of INMT and SEPSECS was favourable for HCC patients. Then, 365 TCGA HCC patients were divided into high-risk and low-risk groups according to the optimal cut-off value, which was determined by X-tile software. The OS of patients in the high-risk group was worse than that in the low-risk group (*p* < 0.001, hazard ratio (HR) = 2.186; 95% CI = 1.549–3.087; Fig. 2A). The expression of INMT and SEPSECS and the distribution of risk scores were also analysed (Fig. 2B). The gene expression of INMT and SEPSECS was significantly negatively correlated with the risk score (Fig. 2B). The prognostic value of this selenium metabolism model was assessed by calculating the area under the curve (AUC) (Fig. 2C). The 1- and 3-year AUCs of the selenium metabolism gene model were 0.691 and 0.641, respectively (Fig. 2C), suggesting that the selenium metabolism model has good accuracy for predicting prognosis. Principal component analysis (PCA) of the selenium metabolism model further validated the grouping ability of INMT and SEPSECS, which can clearly be used to distinguish between low-risk patients and high-risk patients (Supplementary 2A).

### Validation and assessment of the selenium metabolism model in the validation cohort

A validation cohort (LIRI-JP) was used to validate the robustness of the selenium metabolism model. By applying the same equation and the same cut-off value, 228 patients with LIHC in the validation cohort were classified into high-risk and low-risk groups. Similar to the results for the training cohort, patients in the low-risk group had a better survival time than patients in the high-risk group (*P* < 0.001; HR = 2.768; 95% CI = 1.492–5.137) (Fig. 2D). Figure 2E also displays the distribution of the risk score and the expression of INMT and SEPSECS for each patient, which showed a similar tendency as with patients in the training cohort. Similarly, the expression of INMT and SEPSECS was negatively related to the risk score (Fig. 2E). The receiver operating characteristic (ROC) curve showed that the 1- and 3-year AUCs of the selenium metabolism model reached 0.704 and 0.685, respectively (Fig. 2F). These results indicated that the model performed well in predicting the 1- and 3-year prognosis of LIHC patients. Moreover, PCA also displayed the grouping ability of INMT and SEPSECS (Supplementary 2B).

### Prognosis analysis of the selenium metabolism model

**Fig. 3:**
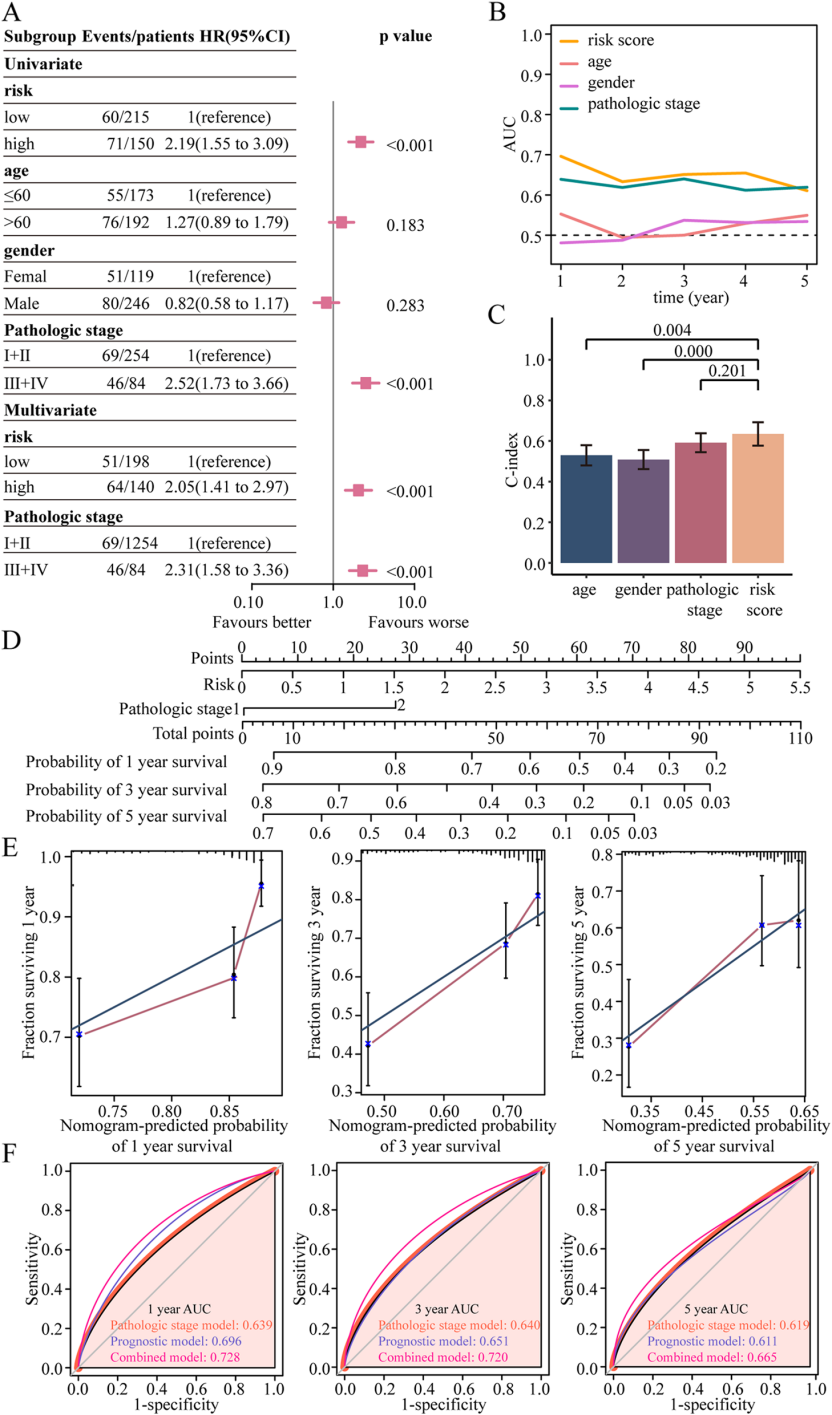
Evaluation of the selenium metabolism gene-mediated model in the training cohort. **A** Univariate and multivariate regression analyses of the selenium metabolism gene-mediated model and clinical characteristics in the TCGA training cohort. Only factors with p values less than 0.05 in univariate analysis were included in multivariate analysis. **B** Comparison of the AUCs of the selenium metabolism model and clinical characteristics such as age, sex and pathological stage. **C** Comparison of the C-indexes of the selenium metabolism gene-mediated model and clinical characteristics such as age, sex and pathological stage. **D** A nomogram for predicting the 1-, 3-, and 5-year survival probabilities was constructed by using the selenium metabolism model and pathological stage. **E** Calibration curves of the nomogram. **F** Comparison of the AUCs of the selenium metabolism gene-mediated model, pathological stage, and nomogram at 1, 3, and 5 years

To understand the role of the selenium metabolism model in the prognosis of HCC patients, we conducted univariate and multivariate Cox analyses. The training cohort (Fig. 3A) and the validation cohort (Supplementary Fig. 2C) showed that the selenium metabolism model was an independent factor for predicting the prognosis of HCC patients. To better evaluate the susceptibility and uniqueness of this model in predicting prognosis, the AUC and concordance index (C-index) of this model were estimated. The AUC of the model was better than that of the other clinicopathological features (age, sex and pathological stage) in the training cohort (Fig. 3B). In the validation cohort, the AUC of the risk score model also tended to be higher than that of the other three clinicopathological features (Supplementary Fig. 2D). The C-index of the selenium metabolism model was higher than those of age and sex in the training cohort (Fig. 3C). However, there was no significant difference in the C-index between the selenium metabolism model and pathological stage (Fig. 3C). In the validation cohort, the C-index of the selenium metabolism model was better than that of age and was not significantly different from that of the other features (sex and pathological stage) (Supplementary Fig. 2E). Overall, these results indicated that the selenium metabolism model could better predict the prognosis of LIHC patients.

### Establishment and validation of a selenium metabolism nomogram

To facilitate the use of the selenium metabolism signature, we developed a nomogram to predict 1-, 3-, and 5-year survival probabilities based on the training cohort. As the selenium metabolism model and pathological stage were two independent factors for predicting prognosis, a nomogram was constructed based on these two factors (Fig. 3D). We calculated the total nomogram score from the sum of the individual scores of those two factors. The higher the patient’s total score was, the worse his or her prognosis. Calibration curves showed that the observed versus predicted 1-, 3-, and 5-year survival probabilities had goodness-of-fit (Fig. 3E). The AUC of the nomogram was better than that of the selenium metabolism gene model and pathological stage alone for predicting 1-, 3-, and 5-year survival (Fig. 3F). All these findings indicated that the nomogram compared with the individual factors was the optimum model for predicting survival.

### Comprehensive analyses of the immune landscape between different risk groups

**Fig. 4:**
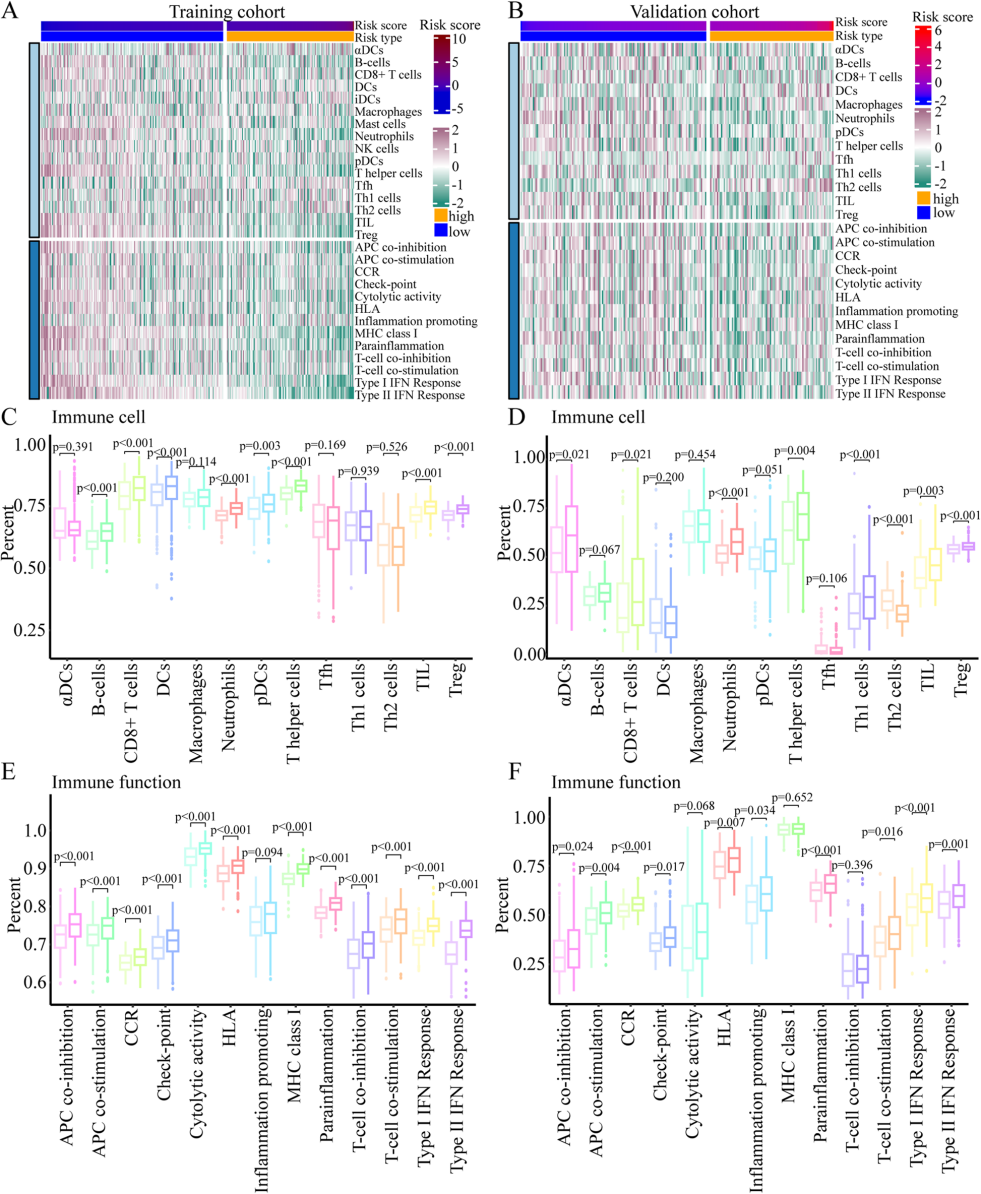
Comprehensive analysis of the differences in immune cell infiltration between high- and low-risk patients in both the training and validation cohorts. **A** and **B** Heatmap of immune cells and immune functions between high-risk and low-risk patients in the training cohort (**A**) and the validation cohort (**B**). **C** and **D**) Comparison of immune cells based on the ssGSEA method between high-risk and low-risk patients in the training cohort (**C**) and the validation cohort (**D**). For each cell type, the left represents high-risk patients, and the right represents low-risk patients. The P value is shown at the top of the graph. **E** and **F** Comparison of immune functions based on the ssGSEA method between high-risk and low-risk patients in the training cohort (**E**) and the validation cohort (**F**)

The heatmap of immune cells and immune functions revealed that there was more immune cell infiltration and stronger immune functions in the low-risk group in the training cohort (Fig. 4A). The same result was found in the validation cohort (Fig. 4B). According to the ssGSEA algorithm, immune cell infiltration was significantly higher in low-risk patients than in high-risk patients in both the training cohort (Fig. 4C) and the validation cohort (Fig. 4D). To further validate this result, MCP-counter [19] was used to estimate the population abundance of tissue-infiltrating immune and stromal cells. In both the training cohort (Supplementary Fig. 3A) and the validation cohort (Supplementary Fig. 3B), low-risk patients showed more immune cell infiltration than high-risk patients. The corresponding immune function was stronger in the low-risk group than in the high-risk group in both the training cohort (Fig. 4E) and the validation cohort (Fig. 4F). Overall, our results showed that the immune function of low-risk patients was stronger than that of high-risk patients.

**Fig. 5:**
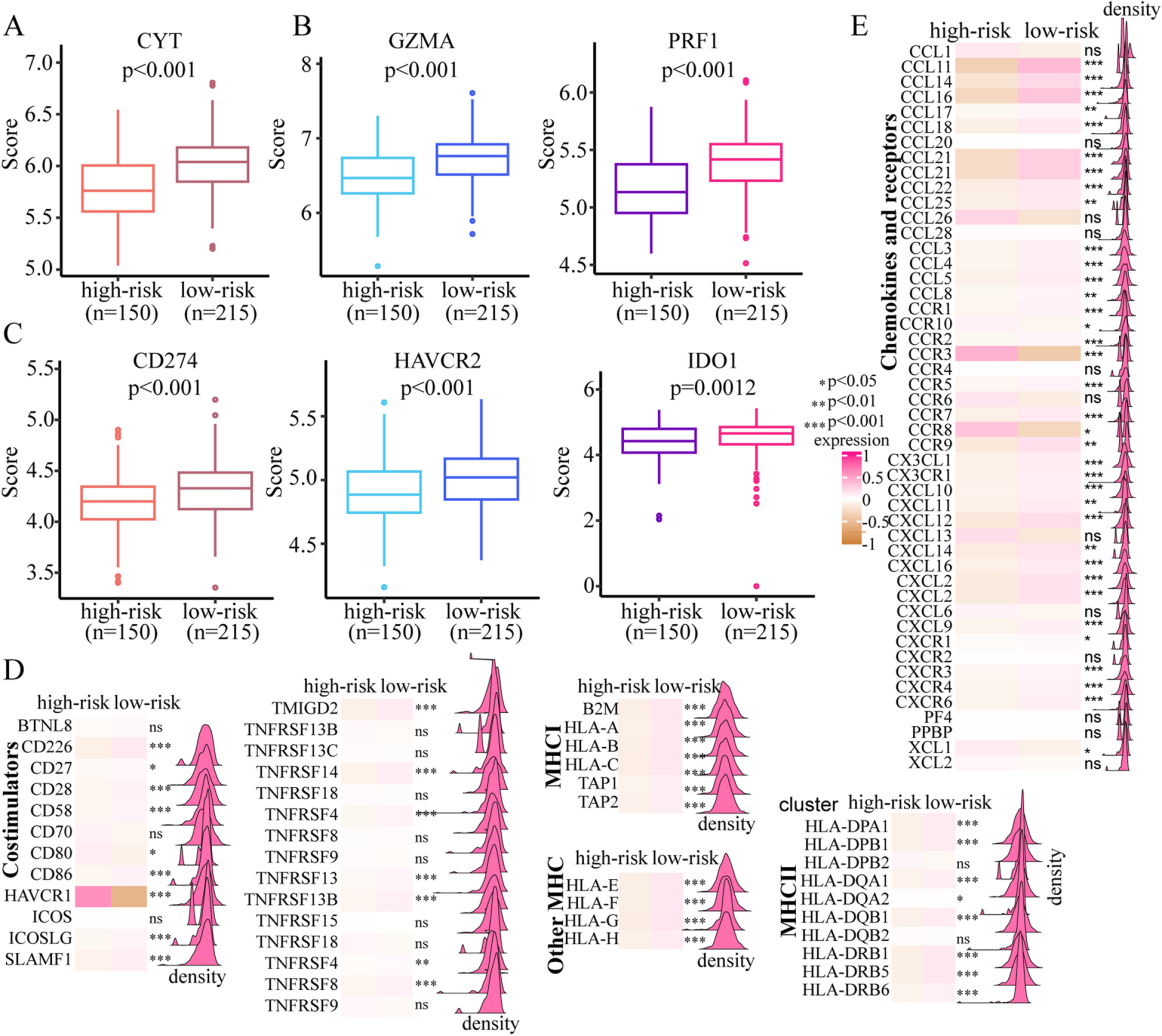
Comprehensive analysis of the differences in immune response genes between high-risk and low-risk patients in the training cohort. **A** and **B** Comparison of the CYT score (**A**), GZMA expression and PRF1 expression (**B**) between high-risk and low-risk patients in the training cohort. **C** Comparison of immune checkpoint expression (CD274, HAVCR2 and IDO1) between high-risk and low-risk patients in the training cohort. **D** Comparison of costimulatory and MHC molecule expression between high-risk and low-risk patients. **E** Comparison of chemokine expression between high-risk and low-risk patients

The CYT score is a prognostic biomarker reflecting host immune status. Next, we compared the CYT score between the two groups. Interestingly, low-risk patients had higher CYT scores than high-risk patients in both the training cohort (Fig. 5A) and the validation cohort (Supplementary 4A). GZMB and PRF1, two key enzymes in cytotoxic T-cell activity, were also significantly higher in the low-risk group than in the high-risk group in both the training cohort (Fig. 5B) and the validation cohort (Supplementary 4B). These results indicate that the immune environment was more conducive to a positive antitumour response in the low-risk group and that higher expression of GZMB and PRF1 in the low-risk group could likely induce more target cell death. Then, we analysed the expression of several immune checkpoints in patients in the two groups. Programmed cell death ligand 1 (PD-L1), hepatitis A virus cellular receptor 2 (HAVCR2) and indoleamine 2,3-dioxygenase 1 (IDO1) were significantly higher in the low-risk group than in the high-risk group in both the training cohort (Fig. 5C) and the validation cohort (Supplementary 4C).

As expected, chemokines and receptors, major histocompatibility complex (MHC) molecules and co-stimulators were significantly higher (most *p* < 0.001) in the low-risk groups in the training cohort (Fig. 5 D and E) and validation cohort (Supplementary 4D and E). Therefore, the low-risk group was more likely to have an immune environment conducive to a positive antitumour response, while the high-risk group was not. We also found that compared to that in patients in the high-risk group, the half-maximal drug inhibitory concentration (IC50) of patients in the low-risk group was significantly lower, which indicated that the low-risk group was more sensitive to rapamycin (*p* = 0.003), sorafenib (*p* < 0.001), metformin (*p* < 0.001) and erlotinib (*p* < 0.001) (Supplementary Fig. 5).

### INMT was downregulated in HCC

**Fig. 6:**
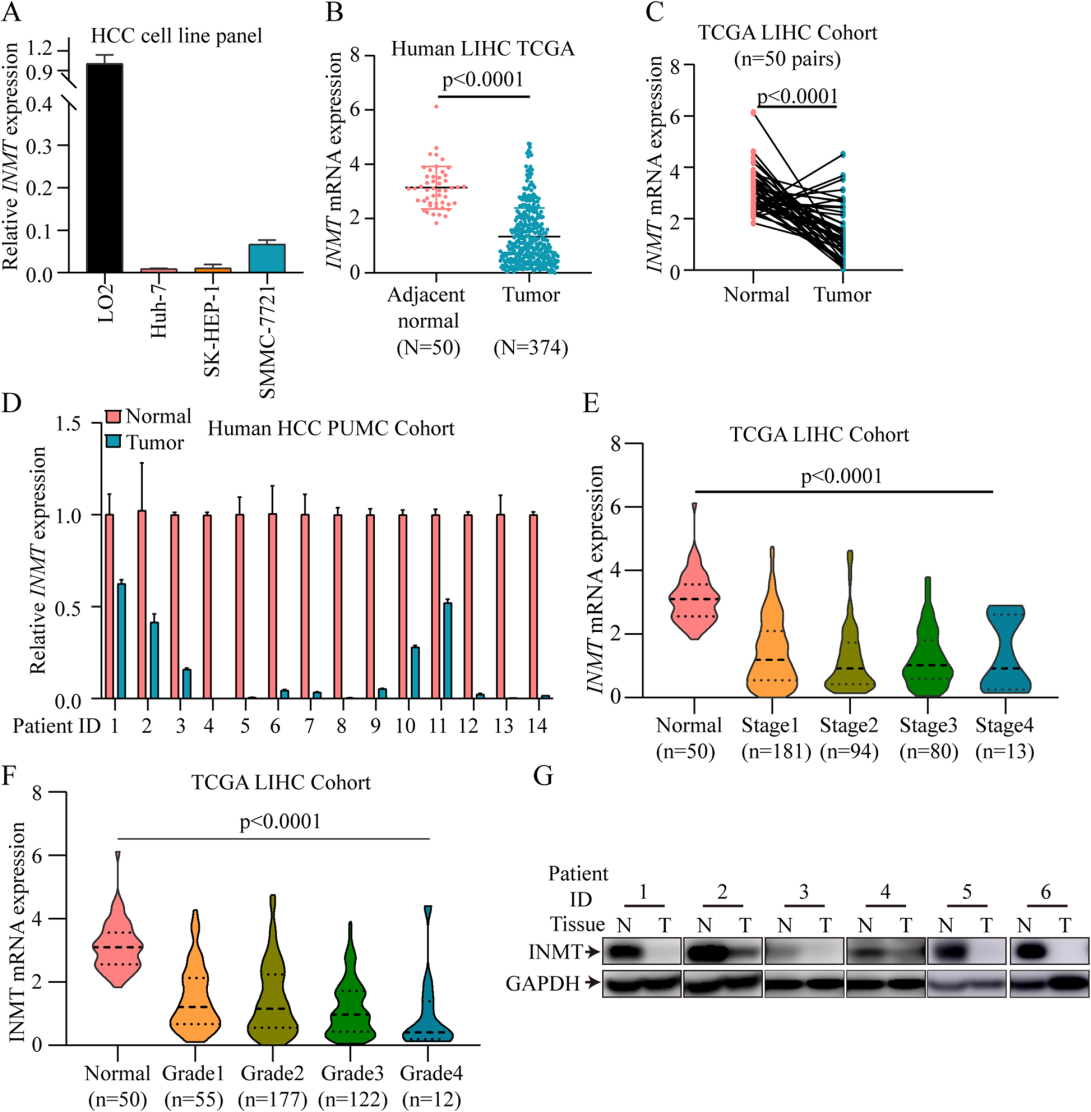
INMT was downregulated in HCC. **A** mRNA expression of INMT in four different types of HCC cells. **B** INMT gene expression in HCC and adjacent normal tissues was analysed in the TCGA liver hepatocellular carcinoma dataset (normal versus tumour). **C** INMT mRNA expression in 50 paired HCC and adjacent normal samples in the TCGA cohort. **D** and **G** INMT gene expression (**D**) and protein expression (**G**) in HCC and adjacent normal tissues were determined in our PUMCH HCC cohort. **E** and **F** The expression features of INMT in samples at different tumour stages (**E**) and with different cancer grades (**F**). HCC, hepatocellular carcinoma

Notably, INMT was significantly downregulated in HCC samples (Fig. 1) and correlated with prognosis (Fig. 2). Therefore, we subsequently focused on the role of INMT in HCC. Initially, we compared the mRNA expression of INMT in different types of liver cells. We found that the mRNA expression of INMT was significantly lower in cancer cells than in LO2 hepatocytes [20] (Fig. 6A). Next, we analysed the mRNA expression of INMT in two independent HCC cohorts (TCGA and our PUMCH cohort). INMT was significantly downregulated in primary HCC compared with adjacent normal tissues in the TCGA cohort (Fig. 6B and C). Its downregulation was also validated by qPCR in our PUMCH cohort (Fig. 6D). INMT protein downregulation was also examined in HCC tissues (Fig. 6G). Next, we examined INMT mRNA expression in six different GEO datasets (Supplementary Fig. 6). In all the datasets, HCC tissues displayed significantly lower expression of INMT than normal tissues. We further evaluated the expression of INMT in different stages or grades of HCC patients (Fig. 6E and F). From early stages (grades) to advanced stages (grades), the expression level of INMT was markedly reduced. We examined the prognostic relevance of INMT (Supplementary Fig. 7). The Kaplan–Meier curve showed that low expression of INMT mRNA was associated with poor survival in LIHC patients (*p* < 0.05). These data indicate that lower expression of INMT is associated with poor prognosis in HCC.

### Knockdown of INMT expression promotes HCC cell proliferation

**Fig. 7:**
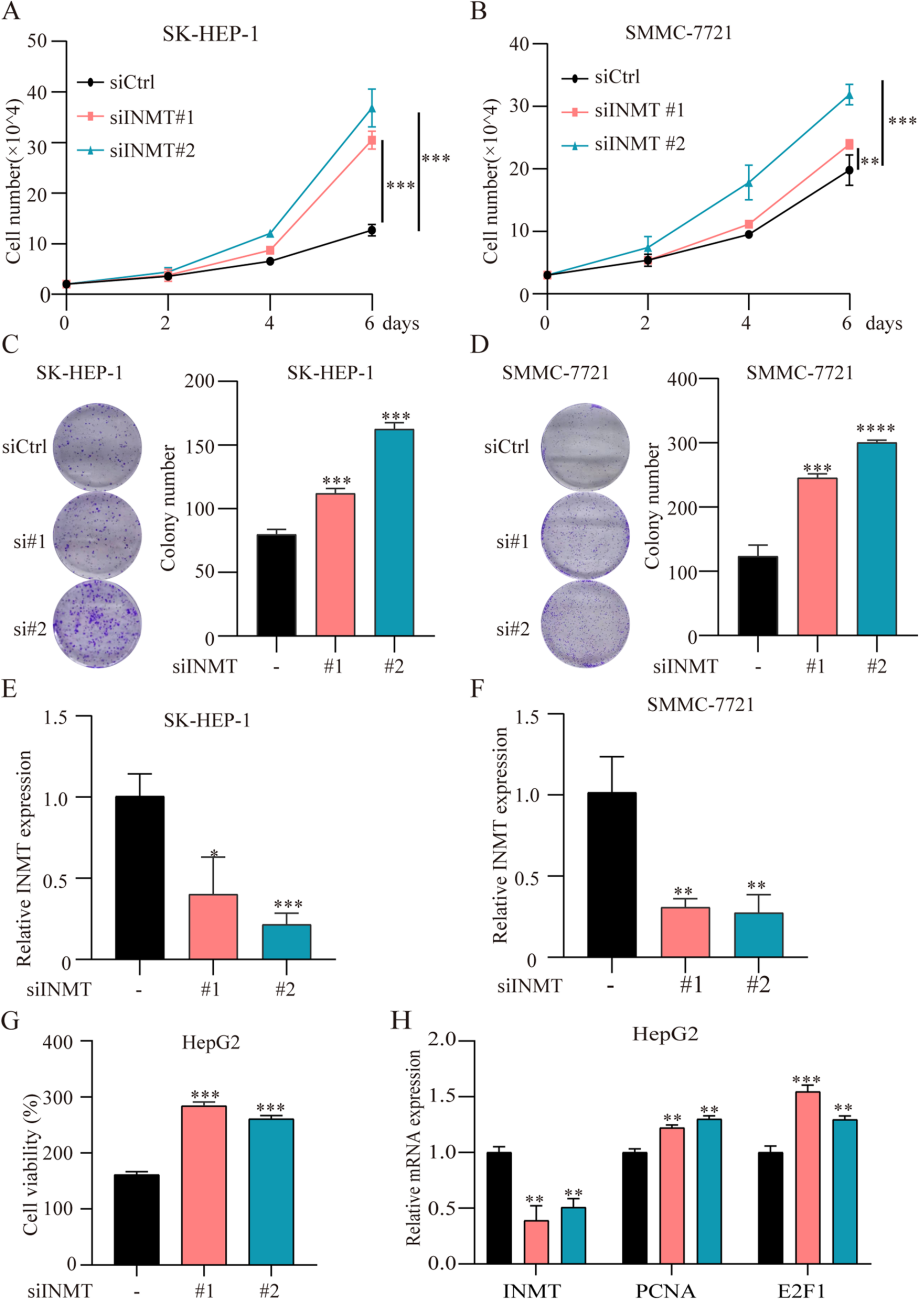
Knockdown of INMT expression promotes HCC cell proliferation. **A** and **C** SK-HEP-1 cells were transfected with control siRNA and INMT siRNA. Cell proliferation (**A**) and colony formation (**C**) are shown. **B** and **D** SMMC-7721 cells were transfected with control siRNA and INMT siRNA. Cell proliferation (**B**) and colony formation (**D**) are shown. **E** and **F** The mRNA expression of INMT in SK-HEP-1 (**E**) and SMMC-7721 (**F**) cells transfected with control siRNA and INMT siRNA. **G** CCK-8 assay of HepG2 cells treated with control siRNA and INMT siRNA. **H** mRNA expression of PCNA and E2F1 in HepG2 cells transfected with control siRNA and INMT siRNA. *P < 0.05, **P < 0.01, ***P < 0.001, ****P < 0.0001

To further explore the role of INMT in HCC, we knocked down INMT using two different sets of small interfering RNAs (siRNAs) in three different types of HCC cells, SMMC-7721, SK-HEP-1, and HepG2 (Fig. 7E, F and H). INMT deficiency led to increased cell proliferation (Fig. 7A, B, and G). Similarly, loss of INMT enhanced cell colony formation (Fig. 7C and D). Cell proliferation markers PCNA and E2F1 were significantly higher expressed after INMT knockdown (Fig. 7H). Taken together, these results suggest that low expression of INMT promotes HCC cell proliferation.

## Discussion

Tumorigenesis is dependent on the reprogramming of cellular metabolism [6]. Altered tumour metabolism reprogramming has profound effects on gene expression and the tumour environment [6]. Selenium is an essential trace element in humans. Inadequate amounts of selenium or selenium deficiency has been linked to an elevated risk of disease, especially cancer [21]. Thus, the regulated whole-body pool of selenium and selenium metabolism are extremely important. Selenoproteins are the most important factors involved in selenium metabolism. Almost 25 human genes encode selenoproteins [22], and nearly all selenoproteins are redox enzymes and glutathione peroxidases that protect cells against oxidative injury [23]. According to the selenocysteine biosynthesis pathway [9], three key enzymes, SEPHS2, PSTK, and SEPSECS, were analysed; moreover, four selenoproteins, GPX1, TXNRD1, GPX4, and SELH, were included in this study. In addition to selenoproteins, excess selenium can be transformed to trimethylselenonium ions (TMSe) through methylation regulation [24, 25]. Therefore, two methyltransferases, INMT and TPMT [10], were analysed in our study.

The liver is the body’s largest metabolism and detoxification organ and is known as the main target organ of selenium deficiency [26]. The liver is also the most important organ regulating the metabolism of carbohydrates, proteins, and other lipids to maintain nutrient balance [27]. Moreover, the liver is sensitive to dietary selenium [28] and is the central organ for selenium regulation and maintaining selenium balance. Liver cancer is a highly malignant form of cancer characterized by altered metabolism [29]. Higher selenium concentrations have been reported to be associated with a significantly lower HCC risk, and the liver appears to be particularly sensitive to selenium supply [30]. Selenium status and selenium intake are negatively associated with hepatitis, cirrhosis, and liver cancer [31]. Yu et al. also found that selenium supplementation protects hepatitis B antigen carriers against the development of HCC [32]. Recently, selenium nanoparticles combined with sorafenib were shown to inhibit HCC progression [33]. The selenium compound CU27 inhibits cancer stem cell generation and reduces primary tumour size in liver cancer [34]. These studies indicate a protective role of selenium in liver cancer. Higher concentrations of selenium may protect against HCC progression. However, at present, the exact role of selenium metabolism regulators in HCC has not been fully reported. Our study focused on the role of the 9 selenium regulators in the TCGA LIHC dataset. Based on the expression profile, we further constructed a selenium metabolism model for the first time. Only two regulators, INMT and SEPSECS, were identified in this model. This model performed well both in the training cohort and in the validation cohort. This model could also predict prognosis of liver cancer patients in both cohorts.

INMT and TPMT are two critical human methyltransferases in selenium methylation. TPMT catalyses the conversion of nonmethylated selenium to the monomethylated form [25]. TPMT expression is reported to be different between normal and pancreatic cancer cells, and knockdown of TPMT sensitizes Panc1 cells to 6-thioguanine [35]. Low TPMT activity is associated with severe toxicity in acute lymphoblastic leukaemia patients [36]. INMT, also named aromatic N-methyltransferase, can transfer one or more methyl groups from S-adenosylmethionine (SAM) to substrates and subsequently create S-adenosylhomocysteine (SAH) [37]. SAM is abundant in the liver, but injury and HCC occur if the level of SAM is chronically excessive [38]. Increasing SAH clearance through the activation of flux via methionine metabolism can extend the lifespan by impacting SAM/SAH levels [39]. Therefore, cells must maintain low concentrations of SAH. Deregulation of INMT expression in primary lung cancer and prostate cancer has been reported [40–42]. Moreover, the expression of INMT has been found to be associated with castration-resistant prostate cancer [43]. In our study, we demonstrate that the expression of INMT is lower in liver cancer, which is consistent with the finding of Carlos and his colleagues [44]. Knockdown of INMT significantly promoted liver cancer cell proliferation and colony formation. The expression of the proliferation-related markers PCNA and E2F1 was significantly elevated after INMT knockdown. All these results indicate that INMT may function as a tumour suppressor in HCC and that INMT affects liver cancer cell proliferation. Previous studies [39] revealed that INMT affects tumour cell apoptosis. The training cohort and validation cohort analysis also revealed that high expression of INMT was correlated with low-risk patients. Low-risk patients may have longer survival times than high-risk patients. The TME in the low-risk group was also more conducive to a positive antitumour response than that in the high-risk group. All these studies revealed that the lower expression of INMT may promote tumour progression. Selenium concentration and SAM/SAH levels may also be abnormal in the TME after INMT knockdown. However, whether the role of INMT in HCC is dependent on its function in selenium metabolism should be further investigated and explained.

SEPSECS catalyses the final step of selenocysteine formation [45]. SEPSECS influences human acute myeloid leukaemia progression [46]. Moreover, Jia Yi et al. reported that SEPSECS expression is decreased in HCC, which is consistent with our findings [47]. Downregulation of SEPSECS may result in the accumulation of selenium and a decrease in selenoproteins. Selenoproteins, such as the GPx family, play an important role in maintaining cellular redox homeostasis and protecting cancer cells against ferroptosis or chemotherapeutic drugs [48, 49].

The prognosis of HCC patients is poor, and the median OS of patients receiving immunotherapy is less than two years [50]. Therefore, we focused on the 1- and 3-year AUC values of this model. In the training cohort, the 1- and 3-year AUCs of the selenium metabolism gene model were 0.691 and 0.641, respectively. In the validation cohort, the 1- and 3-year AUCs of the selenium metabolism model reached 0.704 and 0.685 respectively. All these results suggested that the selenium metabolism model performed well for predicting the prognosis of HCC patients.

Furthermore, the effect of selenium metabolism regulators on immune cell infiltration in the tumour environment is still unclear. Previous studies have shown that tumour-infiltrating lymphocytes serve as prognostic biomarkers and targets for immunotherapy in HCC [51]. In our research, the difference in immune cells and the corresponding immune function between the two risk groups was significant. Most immune checkpoints, chemokines, MHC molecules and some costimulators were significantly higher in the high-risk group than in the low-risk group. Huang et al. [51] also reported the effects of selenium levels on immune-related diseases, including antiviral immunity, autoimmunity, and chronic inflammatory disorders, which further verified our results.

Recently, the role of selenium in cancer immunology has attracted increasing attention. For T cells, supranutritional selenium suppresses RANKL-expressing osteoclastogenic CD4 T cells [52]. Selenium supplementation increases follicular helper T-cell numbers [53], activated Treg cells [54] and Treg subset CD4^+^CD25^−^FOXP3^+^ Tregs [55]. Selenium nanoparticles combined with aerobic exercise training increases Th1 cytokines in splenocytes [56]. Selenium supplementation may also suppress Th1 cell differentiation [57]. Selenium deficiency reduces the number of CD3^+^ and CD8^+^ T-cell populations [58]. For macrophages, selenium nanoparticles induce M1 macrophage polarization [59] and the expression of the fusion receptors CD47 and CD172α and the adhesion molecules CD54 and ICAM-1. Selenium deficiency enhances the migration of immature dendritic cells (DCs), while high selenium levels inhibit immature DC migration [60]. For mature DCs, low selenium levels impair free migration, and high selenium levels inhibit chemotactic migration [60]. Moreover, selenium deficiency may inhibit DC differentiation by downregulating the expression of CD11c, CD40, CD86, and MHCII [61]. Selenium also regulates the immune function of NK cells. Selenium-containing nanoemulsions effectively potentiate the recognition of cancer cells by NK cells [62]. Selenium-containing nanosystems can deliver pemetrexed to tumour sites and increase the sensitivity of human NSCLC cells to NK cells [63].

This study had several limitations. First, the study did not analyse all selenium-related proteins, which limits the understanding of the role of these proteins in HCC. Second, we only validated the INMT gene in the cell experiments. Other genes, such as SEPSECS, may also be prognostic genes for HCC. Finally, we verified that INMT was a prognostic marker for HCC, which was consistent with the results in The Human Protein Atlas. However, together with these findings, the prognostic significance of INMT in HCC is strongly confirmed.

## Conclusions

In summary, this study systematically evaluated the expression of selenium metabolism regulators in HCC. Then, we developed a novel model based on these regulators to predict the prognosis of LIHC patients. The difference in immune cell infiltration in the tumour environment was also evaluated. More importantly, experiments validated that INMT might be a suppressor of HCC progression. In the future, more investigations should be conducted to further reveal the mechanism of selenium regulation in HCC.

## Supplementary Information

**Additional file 1: Supplementary Figure 1.** Expression profile of the 9 selenium metabolism regulators in 50 paired HCCtumor samples and adjacent normal tissues in the TCGA database. 

**Additional file 2: Supplementary Figure 2.** Evaluation of the selenium metabolism gene-mediated model in the validationcohort.

**Additional file 3: Supplementary Figure 3.** Comparison of immune cells between high-risk and low-risk patients.

**Additional file 4: Supplementary Figure 4.** Comprehensive analysis of the differences in immune response genes betweenhigh-risk and low-risk patients in the validation cohort.

**Additional file 5: Supplementary Figure 5.** Comparison of drug sensitivity between low-risk and high-risk groups.

**Additional file 6: Supplementary Figure 6.** INMT expression in GEO datasets.

**Additional file 7: Supplementary Figure 7.** The prognostic significance of INMT expression in HCC.

## Data Availability

All analysed data are included in this published article and its supplementary information file. The original data are available upon reasonable request to the corresponding author.

## Abbreviations

AUC: Area under the curve
C-index: Concordance index
CYT: Cytolytic activity
GEO: Gene expression omnibus
GPX1: Glutathione peroxidase 1
GPX4: Phospholipid hydroperoxide glutathione peroxidase
GZMA: Granzyme A
HAVCR2: Hepatitis A virus cellular receptor 2
HCC: Hepatocellular carcinoma
HR: Hazard ratio
IDO1: Indoleamine 2,3-dioxygenase 1
IC50: The half-maximal drug inhibitory concentration
INMT: Indolethylamine n-methyltransferase
LASSO: Least absolute shrinkage and selection operator
LIHC: Liver hepatocellular carcinoma
LIRI-JP: Liver Cancer-RIKEN-Japan
MCP: Microenvironment Cell Populations-counter
MHC: Major histocompatibility complex
OS: Overall survival
PCA: Principal component analysis
PD-L1: Programmed cell death ligand 1
PRF1: Perforin 1
PSTK: L-seryl-trna(sec) kinase
PUMC: Peking Union Medical College
PUMCH: Peking Union Medical College Hospital
qPCR: Quantitative PCR
ROC: Receiver operating characteristic curve
SAM: S-adenosylmethionine
SAH: S-adenosylhomocysteine
SEPHS2: Selenide, water dikinase 2
siRNAs: Small interfering RNAs
SELH: Selenoprotein h
SEPSECS: O-phosphosery-trna (sec) selenium transferase
ssGSEA: Single-sample gene set enrichment analysis
TCGA: The Cancer Genome Atlas
TMSe: Trimethylselenonium ions
TPMT: Thiopurine s-methyltransferase
TXNRD1: Thioredoxin reductase 1
