# Supplementary figures and images for: Development and validation of a selenium metabolism regulators associated prognostic model for hepatocellular carcinoma
Source: BMC Cancer. 2023 May 18;23:451. doi: 10.1186/s12885-023-10944-w (PMC10197375; doi:10.1186/s12885-023-10944-w)

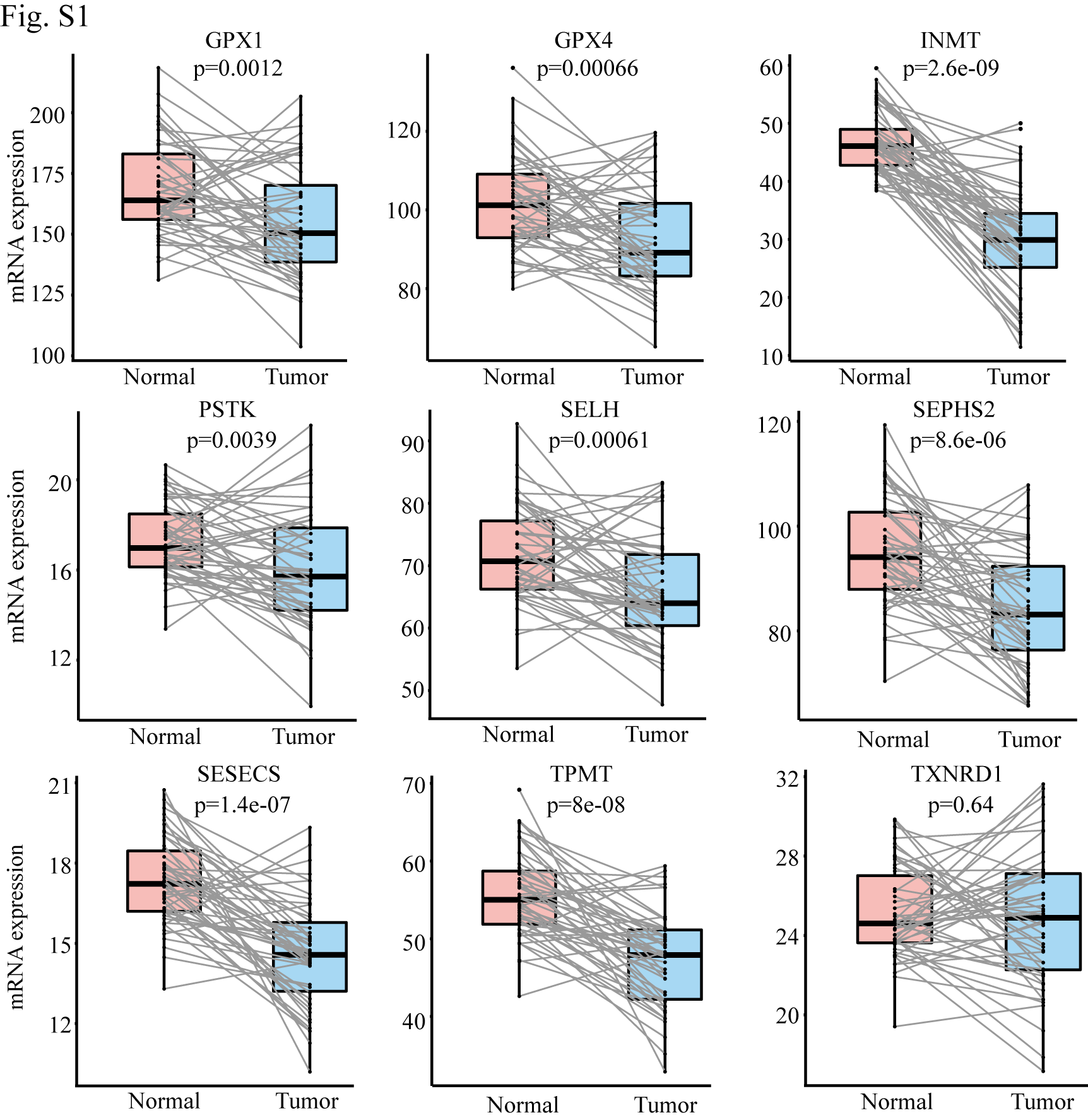

Supplement: Supplementary file 1 — Additional file 1: Supplementary Figure 1. Expression profile of the 9 selenium metabolism regulators in 50 paired HCCtumor samples and adjacent normal tissues in the TCGA database. [file 12885_2023_10944_MOESM1_ESM.tif]

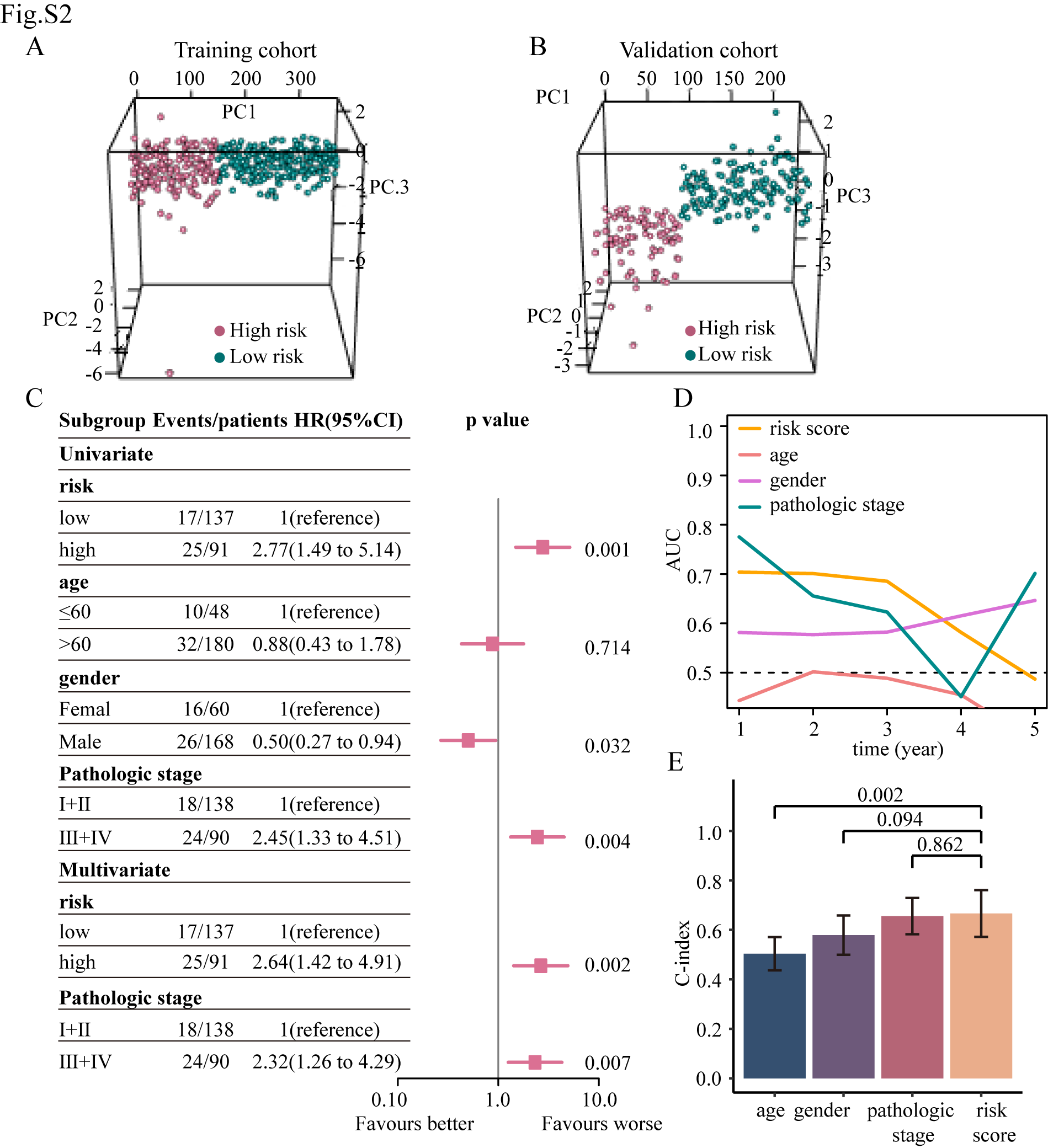

Supplement: Supplementary file 2 — Additional file 2: Supplementary Figure 2. Evaluation of the selenium metabolism gene-mediated model in the validationcohort. [file 12885_2023_10944_MOESM2_ESM.tif]

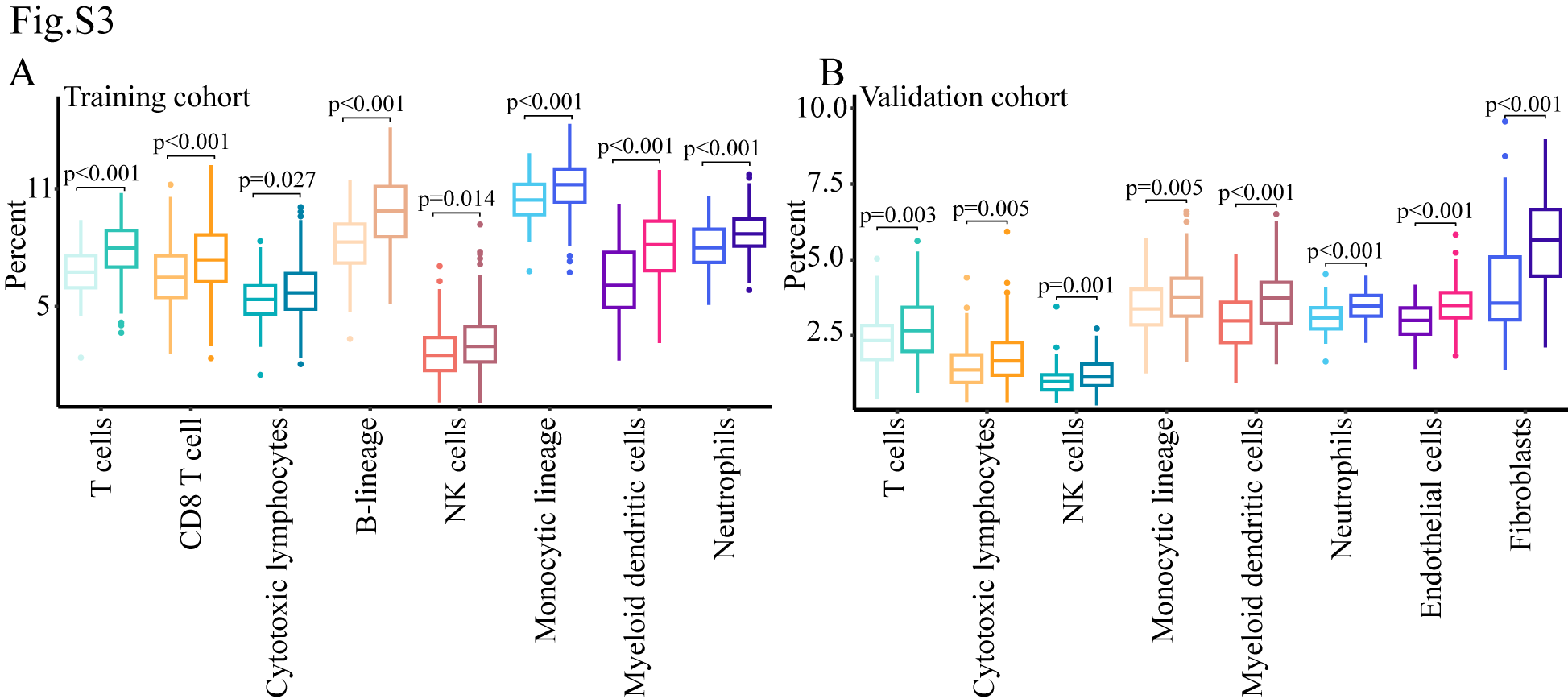

Supplement: Supplementary file 3 — Additional file 3: Supplementary Figure 3. Comparison of immune cells between high-risk and low-risk patients. [file 12885_2023_10944_MOESM3_ESM.tif]

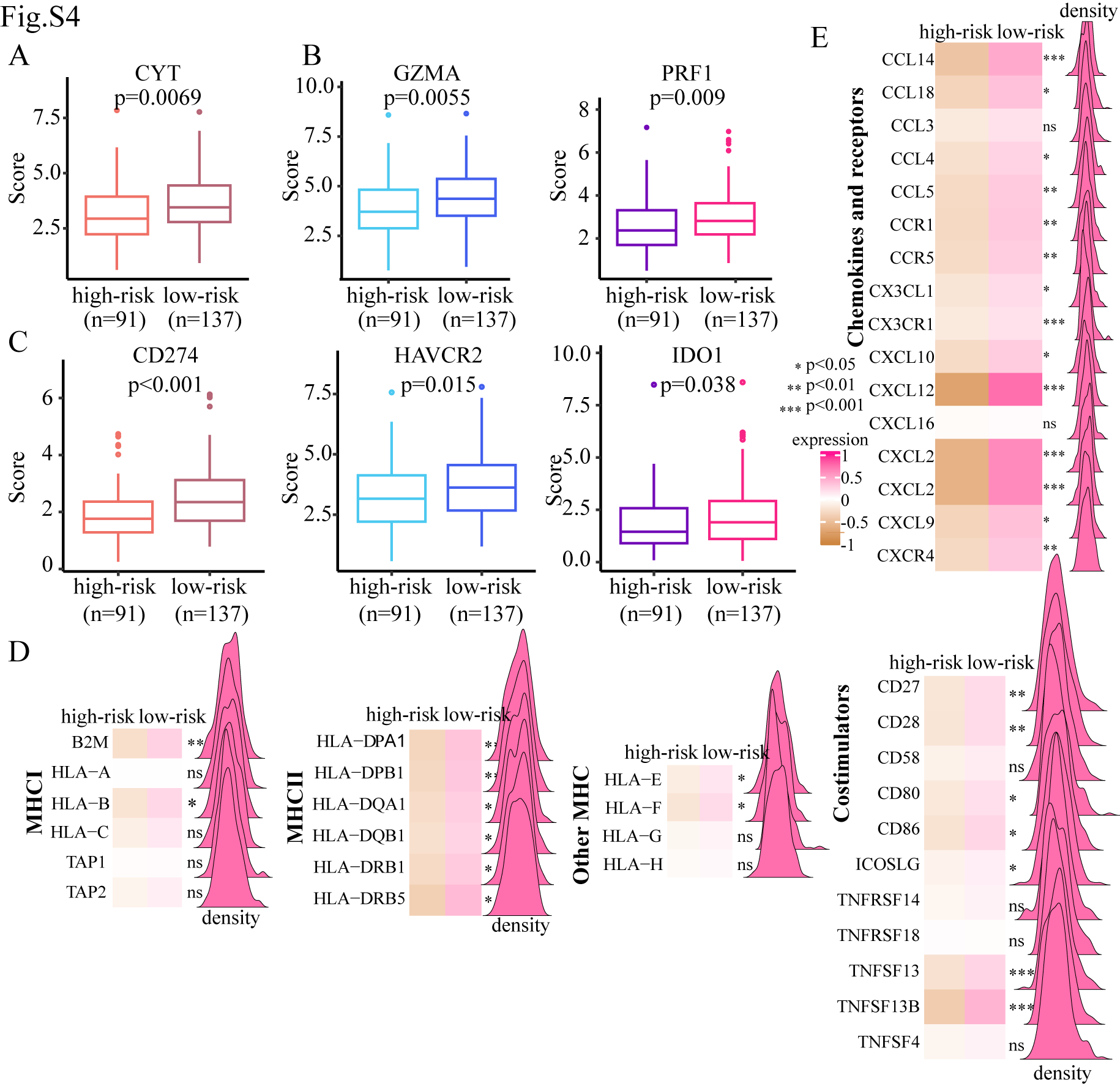

Supplement: Supplementary file 4 — Additional file 4: Supplementary Figure 4. Comprehensive analysis of the differences in immune response genes betweenhigh-risk and low-risk patients in the validation cohort. [file 12885_2023_10944_MOESM4_ESM.tif]

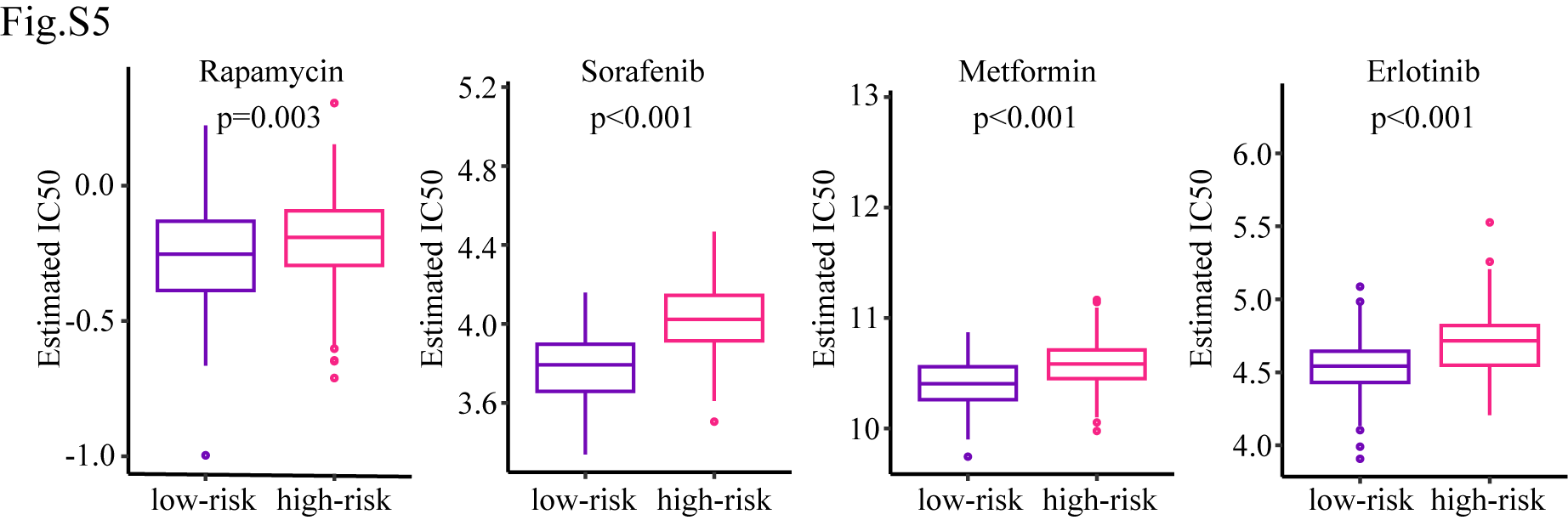

Supplement: Supplementary file 5 — Additional file 5: Supplementary Figure 5. Comparison of drug sensitivity between low-risk and high-risk groups. [file 12885_2023_10944_MOESM5_ESM.tif]

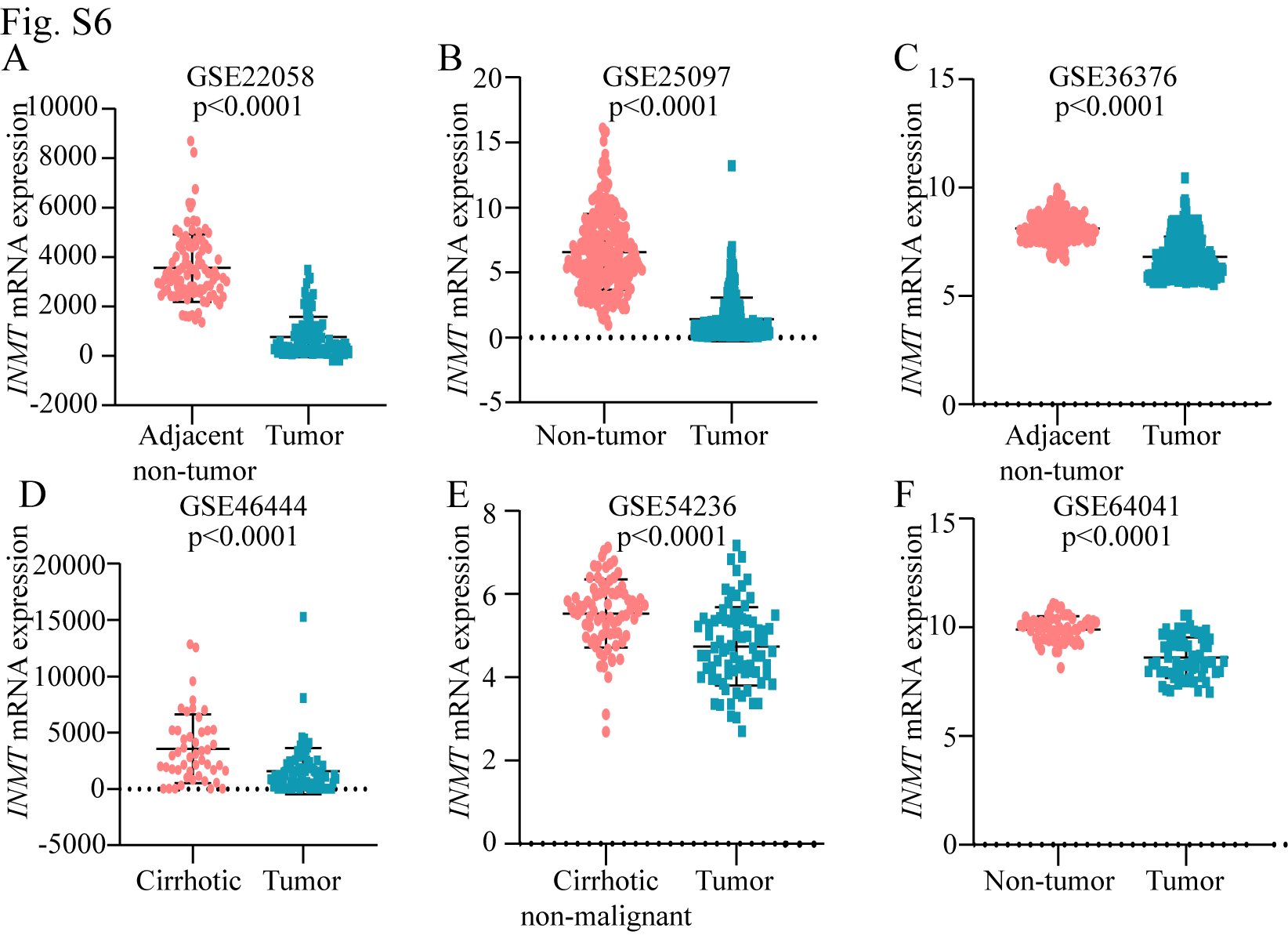

Supplement: Supplementary file 6 — Additional file 6: Supplementary Figure 6. INMT expression in GEO datasets. [file 12885_2023_10944_MOESM6_ESM.tif]

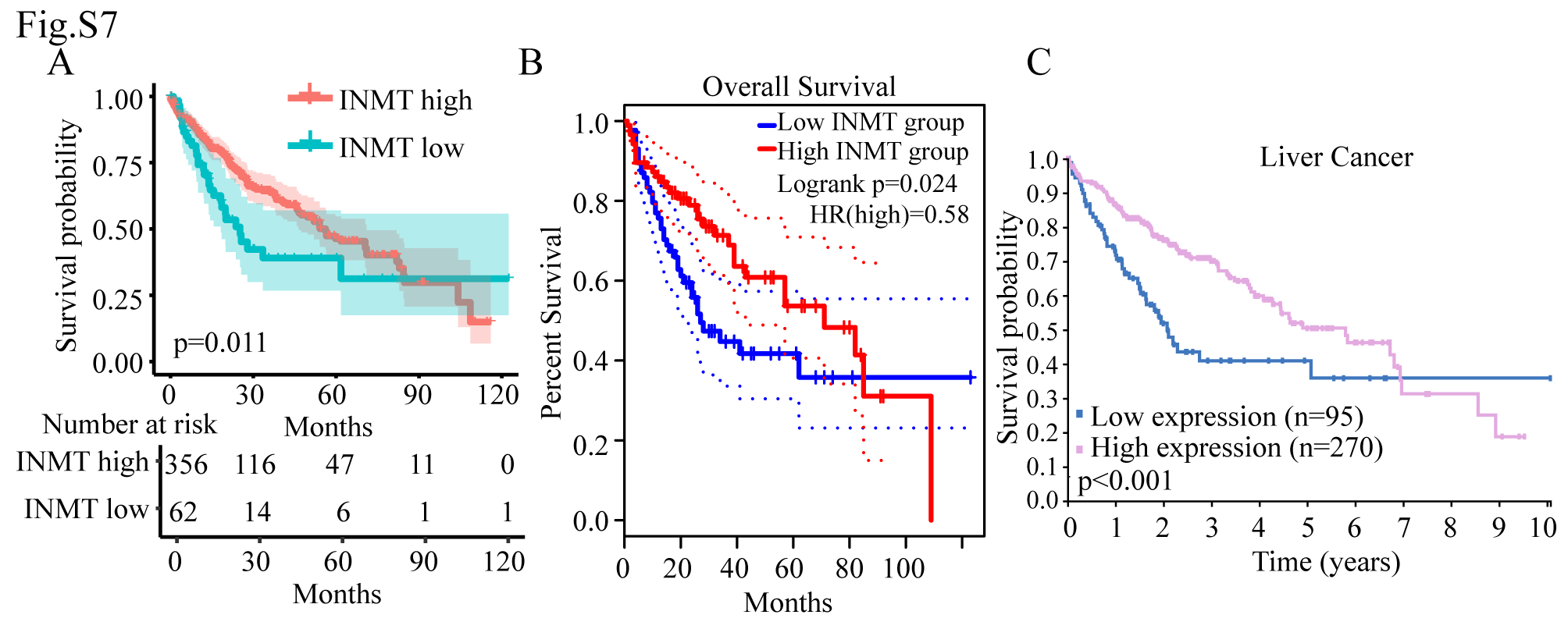

Supplement: Supplementary file 7 — Additional file 7: Supplementary Figure 7. The prognostic significance of INMT expression in HCC. [file 12885_2023_10944_MOESM7_ESM.tif]
